# Different Polymorphisms of *Vascular Endothelial Growth Factor* Gene in Patients with Pre-Eclampsia among The Iranian Women Population

**DOI:** 10.22074/ijfs.2020.5787

**Published:** 2020-02-25

**Authors:** Rana Niktalab, Zeinab Piravar, Roudabeh Behzadi

**Affiliations:** Department of Biology, Faculty of Sciences, Central Tehran Branch, Islamic Azad University, Tehran, Iran

**Keywords:** Iranian Women, Pre-Eclampsia, Single Nucleotide Polymorphism, Vascular Endothelial Growth Factor

## Abstract

**Background:**

Pre-eclampsia (PE) is a pregnancy complication and one of the leading causes of maternal and neonatal morbidity and mortality in the world. PE is characterized by high blood pressure and signs of damage to the other organs, most often the liver and kidneys. Given the importance of mutation in the vascular endothelial growth factor (VEGF) gene and its correlation with the incidence of PE, the relationship of VEGF encoding gene polymorphisms rs922583280, rs3025040 and rs10434 with the incidence of PE in the population of Iranian women was studied, in this research.

**Materials and Methods:**

In this case-control study, 100 pregnant women with PE diagnosis and 50 healthy pregnant women were evaluated using Sanger sequencing method to determine genotypes rs922583280, rs3025040 and rs10434.

**Results:**

There was no significant difference in the allele frequency of rs922583280 and rs3025040 polymorphisms between case and control groups (P>0.05), while frequency of the recessive allele (G) for rs10434 polymorphism was significantly higher in the case group compared to the control group (P=0.014, case=24%, control=12%). Frequency of the allele A in the control group was higher than the patient group (case=76%, control=88%). Frequency of AG genotype in the patient group was also higher than the control group. In addition, frequency of AA genotype in the control group was higher than the patient group (case=57%, control=78).

**Conclusion:**

The results of this study demonstrated a significant difference between patient and control groups for the VEGF coding gene polymorphism rs10434 and it can affect the incidence of PE among Iranian women.

## Introduction

Pre-eclampsia (PE) is one of the most common types of abnormality in pregnancy associating with increased blood pressure in the second half of pregnancy and proteinuria (protein excretion in the urine) and is considered as one of the three main causes of maternal and fetal mortality and related complications (1). This complication is a systemic disorder and it can cause different complications in the mother such as kidney and liver dysfunction, cerebral edema associated with seizure and affliction to hemolysis, elevated liver enzymes, and a low platelet count (HELLP) syndrome. It can also increase risk of abnormality in the fetus, such as fetal growth restriction, which is considered as one of the most important causes of neonatal mortality (2, 3).

Expulsion of the fetus and placenta from the mother’s body eliminates symptoms of the disease, but complications of the disease can be problematic for the child and mother until the end of life (4).

There are two types of PE: mild and severe. Mild form of PE is diagnosed when pregnancy is greater than 20 weeks, blood pressure is greater than 140 systolic or 90 diastolic, 0.3 g of protein is collected in a 24-hours urine sample or persistent 1+ protein measurement on urine dipstick. There is no other sign of problem in the mother or baby (5). Severe type of PE is characterized by a diastolic blood pressure of 110 mm Hg or more, 2+ or higher proteinuria, high creatinine, increased liver enzymes and headache, oliguria, pulmonary edema, upper abdominal pain, visual impairment and thrombocytopenia (6).

Many studies have pointed the importance of vascular endothelial growth factor (VEGF) gene in the pathogenesis of PE (6-8). VEGF gene (that produces an angiogenic protein) is located on chromosome 6 and it contains 4 exons, playing essential role in normal function of the endothelial cells (9). Findings constantly reported reduced free and accessible amount of biological VEGF in preeclamptic women. Production of direct VEGF inhibitor in response to ischemic placenta, as a characteristic of the disease, is a mechanism which often leads to reduced level of free VEGF (10). All members of VEGA family stimulate cellular response by binding to its tyrosine kinase receptors on the cell surface (11). Single nucleotide polymorphisms (SNPs) are intended as a major genetic source of phenotypic changes within a species. They are considered as important markers that are used in diagnosis of disease (12, 13).

Today, a large number of women are suffering from PE during pregnancy. Unfortunately, the main causes of this disease have not yet been known. It seems that the mutation in the VEGF gene is one of the main causes of this disease (6). The aim of this study was to examine the polymorphisms of *VEGF* gene in women patients. Regarding the importance of this issue, we attempted to obtain enough statistical information to consider SNPs for identifying individuals predisposed to the disease through determining possible mutations associated with PE in the affected individuals.

## Materials and Methods

This study was approves by Ethics committee of Islamic Azad University- Science and research branch (Tehran, Iran, approval number: IR.I-AU.SRB. REC.1397.111).

This is a case-control study in which three SNPs of *VEGF* gene including rs922583280, rs3025040 and rs10434 were examined in 100 pregnant women diagnosed with PE and 50 healthy pregnant women referred to the hospitals in Tehran, between 2017 and 2018 (inclusion criteria consists of pregnant women with no history of hypertension and with average of 110 mm hg systolic and 70 diastolic blood pressures).

Exclusion criteria included history of any cardiovascular disease, metabolic disease, hypertension before pregnancy, smoking of cigarette, chronic hypertension and kidney disease before or during this research (14), since these criteria might cause disorders in our studies due to the interference of gene function. Sanger sequencing method was used to determine genotypes. After completing the consent form by the participants, 5 ml volume of venous blood was taken from qualified individuals in the studied groups and it was divided into two tubes; clotting tube for serum separation and Ethylenediaminetetraacetic acid (EDTA) anticoagulant tube for DNA extraction. Blood samples were stored at -20°C. All samples were evaluated using similar methods and conditions. DNA was extracted from all samples using a salting out method. DNA purity and quantity were determined using a Nanodrop 2000 spectrometer (Termo-nanodrop 2000cUSA). Primers were designed using Primer blast software (http:// www.ncbi.nlm.nih.gov/tools/primer-blast). The primers and size of the amplified sequence are mentioned in Table 1.

**Table 1:** The primers and size of the amplified sequences


Gene	Primer sequences (5ˊ-3ˊ)	PCRproduct size

*VEGF*	F: TGGTGAAGTTCATGGATGTCTATC	115
	R: ACACAGGATGGCTTGAAGATG	
	F: GTGCTAATGTTATTGGTGTCTTC	508
	R: CAATGTGTCTCTTCTCTTCGC	


PCR; Polymerase chain reaction

Polymerase chain reaction (PCR) reaction was performed in 25 μl volume, containing 100-300 ng of extracted DNA, 1X PCR buffer (included 50 mM KCl, 10 mM Tris-HCl, 1.5 mM MgCl2), 2 mM MgCl2, 200 µM dNTP mix and double distilled water, 1 U of Taq DNA polymerase (super Taq DNA polymerase, Gen Fanavaran Co., Iran) and 0.4 µM of each oligoneucleotide primer in Thermocycler (Epperndorf-Nexus, Germany). PCR program was performed as follow: enzyme activation at 95ºC for 5 minutes, denaturation at 95ºC for 30 seconds, annealing at 58ºC for 30 seconds, elongation at 72ºC for 30 seconds for 30 cycles and a final extension at 72ºC for 5 minutes. PCR products were loaded on 1% agarose gel followed by ethidium bromide staining to confirm specificity and quality of the amplified fragments (PCR kit, Gen Fanavaran Co. DATA sheet).

To determine genotype of the PCR products, the samples were sequenced. They were next analyzed by FinchTV software and the accuracy of work was ultimately confirmed.

SPSS software (BMI SPSS statistics version 22, USA) was used for data analysis and only 5% was considered as acceptable rate of the type 1 error. The SHEsis software was used to examine the Hardy-Weinberg equilibrium and to evaluate the extent of linkage disequilibrium (LD), D′ and r2 between pairs of polymorphisms. Given the status of data distribution, independent samples t test, Mannwhitney U and one-way ANOVA or Kruskal-Wallis were also used. Odds ratios (OR) with 95% confidence intervals were calculated to determine the odds of developing PE when the individual has gene variants of interest. Comparison of genotype frequencies, association with the disease using the best inheritance model, LD statistics and haplotype analysis, including haplotype frequency estimation, as well as the analysis of association between haplotypes and PE were performed using SNP Stats software. P<0.05 was considered statistically significant.

## Results

The demographic and clinical characteristics of the studied subjects are presented in Table 2. The results of this study showed that there is a significant difference between the groups of patient (case) and control in terms of pregnancy weight gain and blood pressure; so that the weight in the patient group was significantly higher than the control group (P<0.001, OR=2.556).

**Fig 1:**
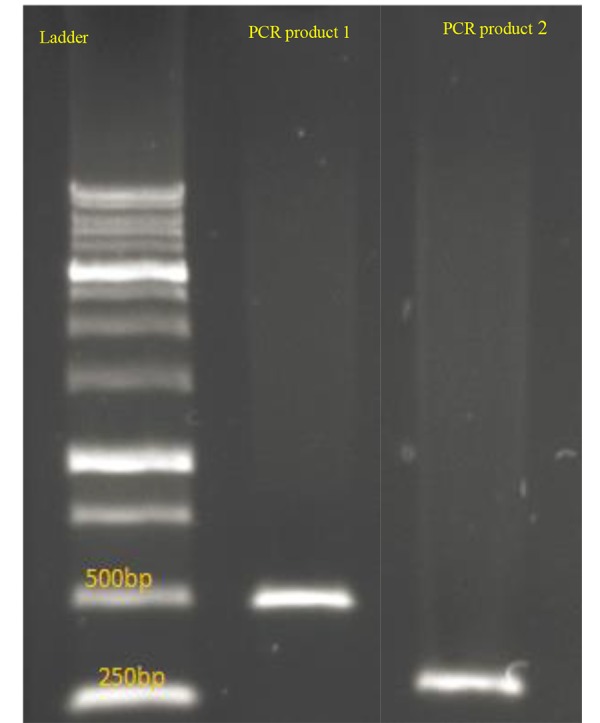
Polymerase chain reaction (PCR) amplification. Fragments of the gene were detected after electrophoresis on 1% agarose gel. Sizes of fragments are 520 bp and 256 bp.

According to this study, it was also found that there is significant difference between systolic (P<0.001) and diastolic blood pressures (P<0.001) of these groups. So that blood pressure in the patient group was higher than the control group (Table 2), but there was no significant difference in BMI (P=0.131, OR=0.575) and age (P=0.217, OR=0.364) between the case and control groups. PCR fragments of this gene were detected after electrophoresis on 1% agarose gel. Sizes of fragments are 520 bp and 256 bp (Fig .1).

We determined which allele combination from the two SNPs associated with preeclampsia (Table 3). Three allele combinations including C-C-A and CC-G haplotypes, with 7-23% frequency, were associated with preeclampsia (Figes.S1-3, See Supplementary Online Information at www.ijfs.ir).

In addition, no significant difference was determined in the frequency of rs922583280 and rs3025040 polymorphisms between the patient and control groups. The frequency of recessive allele G, in the rs10434 polymorphism was significantly higher in the patient group rather than that the control group (case=24, control=12 OR, R=2.316: from 1.167 to 4.594, P=0.014), while the frequency of allele A in the control group was higher than the patient group (case=76, control=88, OR=2.316: from 1.167 to 4.594, P=0.014). The frequency of AG genotypes in the patient group was also higher than control group (OR=2.452: from 1.099 to 5.467, P=0.026), while frequency of AA genotype in the control group was higher than the case group (OR=2.675: from 1.229 to 5.820, P=0.012, Table 4).

The interaction between any possible pair of SNPs was visualized by SHEsis program. Analysis revealed linkage disequilibrium (LD) between rs922583280 and rs3025040 (Dˊ=1.000 and r^2^=0.001), while weak LD was determined between rs922583280 and rs10434 as well as rs3025040 and rs10434 (Dˊ=0.062 and r^2^=0.001; Dˊ=0.299 and r^2^=0.001, respectively, Table 3).

Minor allele frequency for VEGF SNPs were rs10434: A>G (A=0.3476/1741; 1000Genomes), rs3025040: C>T (T=0.1512/757; 1000Genomes), rs922583280 C>T (minor allele frequency is not specified.

**Table 2:** Anthropometry and blood pressure data in the patient (case) and control groups


Characteristics	Case group	Control group	P value

Age (Y)^a^	25.8 (7.16)	24.7 (6.22)	0.217
Gestational age (weeks)^a^	32.9 (4.02)	33.1 (4.71)	0.797
Gestational weight gain (kg)^b^	12.7 (8.50-16.50)	10.0 (6.75-13.55)	0.003^*^
Systolic blood pressure (mmHg)^b^	170 (160.0-180.0)	110 (100.0-120.0)	<0.001^*^
Diastolic blood pressure (mmHg)^b^	110 (100.0-120.0)	70 (70.0-80.0)	<0.001^*^
Body mass index (kg/m^2^)^b^	24.05 (21.63-28.13)	23.35 (20.53-26.90)	0.131


^a^; Characteristics are presented as mean (standard deviation), ^b^; Characteristics are presented as median (ranges), and *; P<0.05: statistic significant.

**Table 3:** Haplotype frequencies of VEGF rs922583280, rs3025040 and rs10434 polymorphisms in case and control groups


Haplotypes	Cases (%)n=100	Controls (%)n=50	P value^a^	OR (95% CI)

*VEGF* rs922583280, rs3025040 and rs10434				
C - C - A	0.070	0.847	0.002	0.365 (0.187-0.712)
C - C - G	0.235	0.103	0.006	2.648 (1.283-5.467)
C - T - A	0.050	0.023	0.274	2.214 (0.514-9.543)


*VEGF*; Vascular endothelial growth factor, OR; Odds ratio, CI; Confidence interval, and ^a^; Evaluated by Pearson’s Chi-squared test.

**Table 4:** Haplotype frequencies of VEGF rs922583280, rs3025040 and rs10434 polymorphisms in case and control groups


Gene	Case group (%)	Control group (%)	P value^a^	OR (95% CI)

Rs922583280				
CC	97.00	98.00	0.593	0.660 (0.067-6.507)
CT	3.00	2.00	0.593	0.660 (0.067-6.507)
TT	0.00	0.00	ND	ND
Frequency of C allele	98.50	99.00	0.722	1.508 (0.155-14.681)
Frequency of T allele	1.50	1.00	0.722	1.508 (0.155-14.681)
Rs3025040				
CC	91.00	92.00	0.552	1.137 (0.332-3.891)
CT	8.00	8.00	1.00	1.00 (0.286-3.495)
TT	1.00	0.00	0.478	0.990 (0.971-1.010)
Frequency of C allele	95.00	96.00	0.102	0.474 (0.190-1.179)
Frequency of T allele	5.00	4.00	0.302	0.605 (0.231-1.585)
Rs10434				
AA	57.00	78.00	0.012^*^	2.675 (1.229-5.820)
AG	38.00	20.00	0.026^*^	2.452 (1.099-5.467)
GG	5.00	2.00	0.377	2.579 (0.29312.690)
Frequency of A allele	76.00	88.00	0.014^*^	2.316 (1.167-4.594)
Frequency of G allele	24.00	12.00	0.014^*^	2.316 (1.167-4.594)


OR; Odds ratio, CI; Confidence interval, ^a^; P<0.05: Statistically significant, and ND; Not defined

**Table 5:** Minor allele frequency and Hardy-Weinberg tests for the study of population


SNP	MAF	HWE P

rs10434	0.3476	0.676
rs3025040	0.1512	0.114
rs922583280	-	0.878


SNP; Single nucleotide polymorphism, MAF; Minor allele frequency, and HWE P; HardyWeinberg equilibrium P value.

## Discussion

Untreated PE causes serious and fatal complications for mother and baby (1). Given the importance of mutations in the *VEGF* gene, their correlation with the incidence of PE and early delivery, and the risk of afflicting to eclampsia and mortality caused by it for mother and fetus, we attempted to identify possible mutations and early genetic detections.

In this study which was carried out on Iranian pregnant women with PE, a total number of 150 pregnant women, including 100 pregnant women diagnosed with PE and 50 healthy pregnant women, were examined. Mean age in the patients group was 25.8 ± 7.16 and in the control group was 24.7 ± 6.22. This difference was not significant. Analysis represented that frequency and distribution of rs10434 polymorphism allele and genotype in both control and case groups showed a significant difference, so that the frequency of recessive allele G in the patient group was significantly higher than the control group.

It was also found that frequency of the allele A in the control group was higher than that of the patient group. In the genotypic frequency study, the results showed that frequency of AG genotype in the patient group was higher than the control group and frequency of AA genotype in the control group was higher than that of the patient group. In the case of rs922583280 and rs3025040, there was no significant difference in the allele and genotype frequency between these groups. The results of our data were similar to the studies conducted on PE patients in other populations, as it was found in the meta-analysis study conducted on four VEGF gene polymorphisms by Song et al. (15). In this study, it was demonstrated that rs2010963 polymorphism was associated with the incidence of PE in Asian and European populations. Moreover, rs3025039 polymorphism was associated with this disease in the Asian population, while rs1570360 and rs699947 polymorphisms had no correlation with the incidence of the disease (16). Similarly, in a study done by Salimi et al. (17) on Iranian population, it was found that rs2010963 polymorphism in the *VEGF* gene was associated with the incidence of PE. In similar studies conducted by Hansen et al. (18) and Chedraui et al. (16), it was found that there was no significant association between the polymorphisms of VEGF gene and the incidence of disease. A notable point in the present study and the studies conducted on different populations is that polymorphisms in the untranslated regions (UTRs) of *VEGF* gene exons are mainly associated with the incidence of disease. Because these regions play an important and vital role in the trimming process, it can be concluded that mutation in these regions can affect function of VEGF and lead to disorders encountered PE. However, this was not observed in some cases, such as rs3025040 polymorphism, which is in the UTR region. The reason of controversial findings in different studies can be due to differences in populations and breeds of these studies, considering the fact that gene polymorphisms are affected by this important factor (19). 

In general, it can be concluded that *VEGF* gene polymorphisms are associated with the incidence of PE in Iranian women. So that, it is concluded in this study that allele G in the polymorphism rs10434 as well as genotype AG in the same polymorphism may lead to the increased incidence of PE in this population, while no relationship of rs3025040 and rs922583280 alleles and genotypes with the incidence of PE was determined. Moreover, there was no significant relationship between anthropometric factors and genotype of all three polymorphisms (rs922583280, rs3025040 and rs10434) in both patient group with PE and control group.

## Conclusion

Investigations showed that frequency and distribution of rs10434 polymorphism alleles and genotypes had significant difference between control and case groups, so that the frequency of recessive allele G in the patient group was significantly higher than the control group. In the genotypic frequency study, the results showed that frequency of AG genotype in the patient group was higher than the control. In the case of the frequency of alleles and genotypes for two polymorphisms rs922583280 and rs3025040, there was no significant difference between patient and control groups. Some of the limitations which can be mentioned here include small size population of the study and lack of the concomitant VEGF level in plasma. Further studies consisting of the larger and classified cohort are needed to validate our initial findings and to determine association of the other clinical variables and SNPs with the subtypes of PE. Moreover, several clinical parameters, including plasma VEGF, PlGF and sFlt-1 levels and polymorphisms of the other VEGF family (PlGF) members should be put into the prospective account.

## Supplementary PDF


