# Supplementary material for: Different Polymorphisms of Vascular Endothelial Growth Factor Gene in Patients with Pre-Eclampsia among The Iranian Women Population
Source: Int J Fertil Steril. 2020 Feb 25;14(1):41–5. doi: 10.22074/ijfs.2020.5787 (PMC7139223; doi:10.22074/ijfs.2020.5787)
Supplement: Supplementary file 1 [file Int-J-Fertil-Steril-14-41-s01.pdf]

Int J Fertil Steril
International Journal of Fertility & Sterility
ISSN:2008-076X eISSN:2008-0778

Supplementary Information for

Different Polymorphisms of *Vascular Endothelial Growth Factor* Gene in Patients with Pre-Eclampsia among The Iranian Women Population

Rana Niktalab, M.Sc., Zeinab Piravar, Ph.D.*, Roudabeh Behzadi, Ph.D.

Department of Biology, Faculty of Sciences, Central Tehran Branch, Islamic Azad University, Tehran, Iran

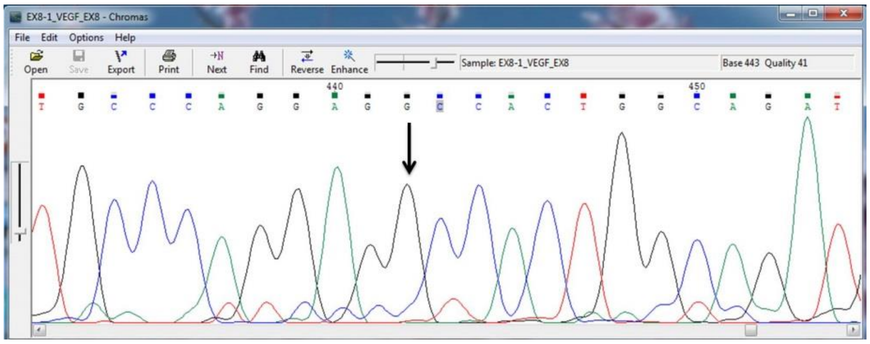

Fig.S1: Polymerase chain reaction (PCR) product sequencing showing the pick and desired allele with GG genotype for rs10434.

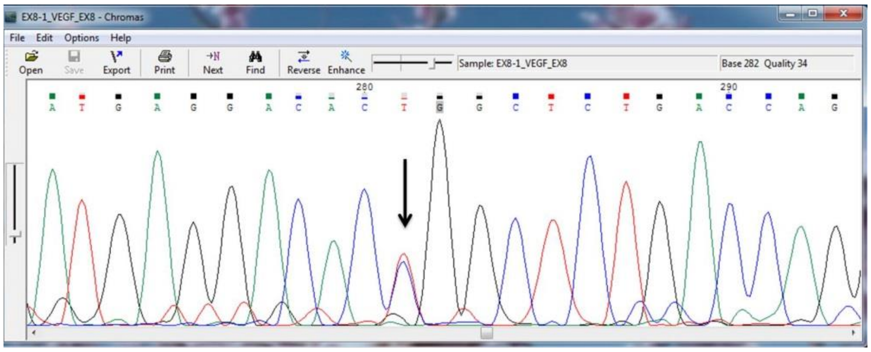

Fig.S2: Polymerase chain reaction (PCR) product sequencing showing the pick and desired allele with CT genotype for rs3025040.

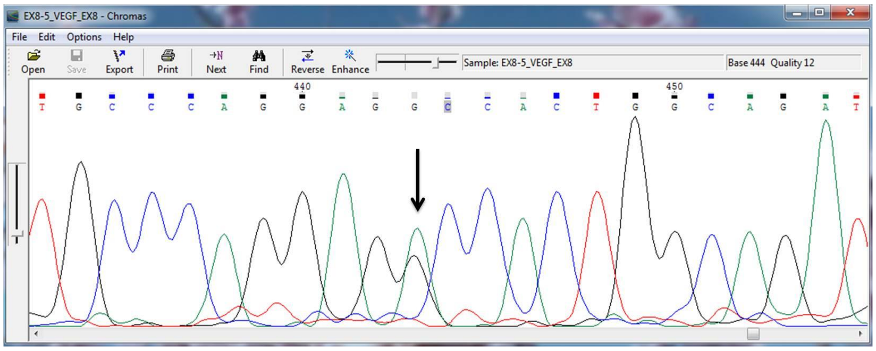

Fig.S3: Sanger sequencing. Sequencing of the polymerase chain reaction (PCR) product amplified for the desired allele with AG genotype (rs10434).

Received: 14/November/2018, Accepted: 26/September/2019
***Corresponding Address: P.O.Box: 13117773591, Department of Biology, Faculty of Sciences, Central Tehran Branch, Islamic Azad University, Tehran, Iran**
Email: saba.piravar@gmail.com
